# Layer-by-Layer Method for the Synthesis and Growth of Surface Mounted Metal-Organic Frameworks (SURMOFs)

**DOI:** 10.3390/ma3021302

**Published:** 2010-02-23

**Authors:** Osama Shekhah

**Affiliations:** Institute of Functional Interfaces, Karlsruhe Institute of Technology, 76344 Karlsruhe, Germany; E-Mail: Osama.Shekhah@ifg.kit.de; Tel.: +49 724 78 26946; Fax: +49 724 78 23478.

**Keywords:** layer-by-layer, SURMOFs, SAMs, thin films

## Abstract

A layer-by-layer method has been developed for the synthesis of metal-organic frameworks (MOFs) and their deposition on functionalized organic surfaces. The approach is based on the sequential immersion of functionalized organic surfaces into solutions of the building blocks of the MOF, *i.e.,* the organic ligand and the inorganic unit. The synthesis and growth of different types of MOFs on substrates with different functionalization, like COOH, OH and pyridine terminated surfaces, were studied and characterized with different surface characterization techniques. A controlled and highly oriented growth of very homogenous films was obtained using this method. The layer-by-layer method offered also the possibility to study the kinetics of film formation in more detail using surface plasmon resonance and quartz crystal microbalance. In addition, this method demonstrates the potential to synthesize new classes of MOFs not accessible by conventional methods. Finally, the controlled growth of MOF thin films is important for many applications like chemical sensors, membranes and related electrodes.

## 1. Introduction

Metal-Organic Frameworks (MOFs) are hybrid inorganic-organic solid compounds with porosity similar to zeolites, but transgressing their limitations in terms of materials design. In general, inorganic coupling units are combined with organic linkers, yielding molecular cages with vertices defined by the ligands and corners defined by the inorganic coupling units [1,2,3]. A large body of research on MOFs is directed to unravel the rules of reticular synthesis and to develop the tool-box needed for the rational “design” of MOFs with desired properties [2,3,4,5]. Whereas the first application proposed for these highly porous materials was the storage of gas molecules, in particular hydrogen [6], the fascinating properties of this new class of materials have inspired a huge variety of other potential applications, including separation [7,8,9], catalysis [7,10,11], drug release [12] and the imbedding of metal-nanoparticles for applications in catalysis and sensor technology [13,14]. Chemical sensors and many other related electro-devices and applications depend on the fabrication of thin films and coatings of defined porosity combined with tuneable chemical functionality.

Zeolites, organic polymers, metal oxides, activated carbon and MOFs were the typical materials to use for this purpose [13]. However, for zeolites and related siliceous materials the range of control of functionality on a molecular level is nevertheless limited. In case of MOFs, this is a difficult task considering the solvothermal synthesis conditions [15,16].

In this work we present a new layer-by-layer method (LBL) we have developed to synthesize and grow MOFs on surfaces (surface-mounted MOFs (SURMOFs)) [17]. This concept is based on surface chemistry and is in principle related to the solid-phase synthesis of complex (bio-)organic polymers, such as peptides, DNA, *etc.*, by using an appropriately functionalized organic surface as a (two-dimensional) nucleation site [18,19]. In contrast to the established synthesis protocols of MOFs in general, where the educts (primary building blocks, typically two) are mixed and treated under solvothermal conditions, the LBL growth mode of MOFs is based on the combination of the reaction partners in a sequential fashion separated by removing unreacted components after each step. Using this route we have been able to synthesize and grow different types of MOFs on organic surfaces with different functionalities.

## 2. Results and Discussion

### 2.1. Synthesis and growth of [Cu_3_(btc)_2_.n(H_2_O)] (HKUST-1) SURMOF

**Figure 1:**
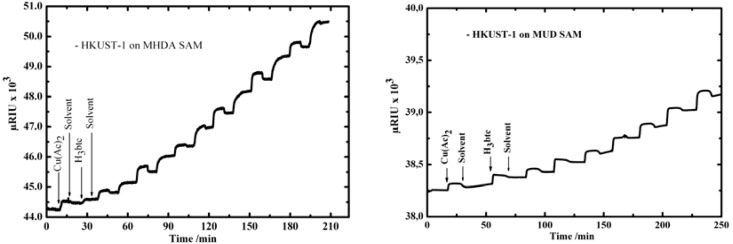
SPR signal as a function of time recorded *in situ* during sequential injections of Cu(Ac)_2_, ethanol, and H_3_btc in the SPR cell containing MHDA SAM (left) and MUD SAM (right) [13,17].

HKUST-1 [Cu_3_(btc)_2_.n(H_2_O)] MOF was the first successful type of MOF investigated and synthesized using the LBL method; whereas the synthesis and structure of this MOF have been described in detail previously [20], still the details of its formation are unknown. In Figure 1 (left) we present data obtained by surface plasmon resonance (SPR) for the growth of HKUST-1 on a COOH-terminated SAM surface fabricated by immersing the Au substrate into an ethanolic solution of 16-mercaptohexadecanoic acid (MHDA). The SPR technique allows monitoring the deposition of molecular species on surfaces with a submonolayer resolution. The data shows that subsequently adding Cu_2_(CH_3_COO)_2_.H_2_O (Cu(Ac)_2_) and 1,3,5-benzenetricarboxylic acid (H_3_btc) leads to a stepwise growth of inorganic/organic multilayers. In addition, infrared reflection absorption spectroscopy (IRRAS) characterization fully support this finding, where a linear increase in the intensity of COO IR bands with the number of immersion cycles was observed.

The growth of HKUST-1 on an OH-terminated SAM surface fabricated by immersing the Au substrate into an ethanolic solution of 11-mercaptoundecanol (MUD) was also investigated, as shown in Figure 1 (right). The SPR and IRRAS data showed the same growth behavior as observed on the MHDA SAM, but interestingly with a growth rate slower than on the MHDA SAM [21].

**Figure 2:**
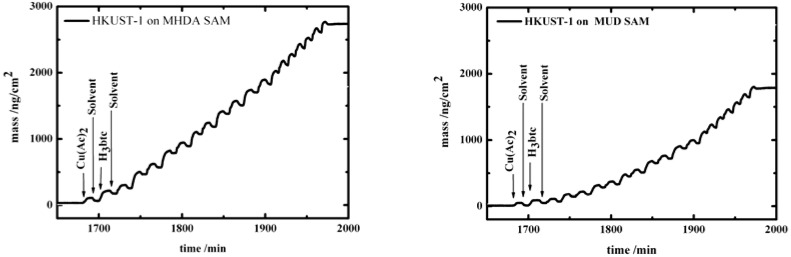
QCM-D signal as a function of time recorded *in situ* during sequential injections of Cu(Ac)_2_, ethanol and H_3_btc on the QCM substrate covered by a MHDA SAM (left), and a MUD SAM (right).

The growth of HKUST-1 on both MHDA and MUD SAMs was also monitored using quartz crystal microbalance with dissipation (QCM-D) (Figure 2), where the mass increase of deposited molecular species on both surfaces can be monitored. The data show that subsequently adding Cu(Ac)_2_ and H_3_btc leads to a stepwise increase of the mass of the deposited layers. The growth rate on the MUD surface Figure 2 (right) was also again found to be slower than on the MHDA surface Figure 2 (left).

**Figure 3:**
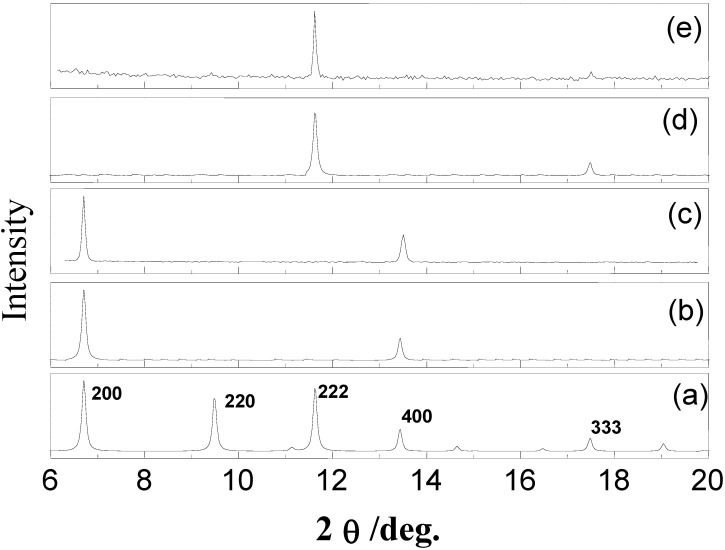
Out-of-plane XRD data for a Cu_3_(btc)_2_.xH_2_O MOF (a) Bulk, (b) growth on a MHDA SAM (simulation), (c) experimental growth on MHDA SAM (experimental), (d) growth on MUD SAM (simulation), (e) grown on MUD SAM (experimental) [21].

The XRD data shown in Figure 3 clearly demonstrates the success of the LBL method. This out-of-plane diffraction scan clearly demonstrates the presence of a highly ordered and preferentially oriented crystalline material. Together with the in-plane data, this demonstrates clearly that the deposited multilayer exhibits the same structure as observed for the bulk compound HKUST-1.

Whereas on a COOH-functionalized surface the growth of HKUST-1 proceeds along the (100) direction, on an OH-terminated surface MOF-layers grow along the (111) orientation. Thus, the role of the organic surface is not only to act as a nucleation template for the MOF growth, but also to control the growth direction. The difference in the growth direction could also explain the difference in the growth rate observed on both surfaces [21]. In this context, we would like to mention that MOF thin films were also grown using the *in situ*-crystallization method, where SAMs were immersed at room temperature into an aged and filtered mother solution of the MOF from the solvothermal synthesis [16,22]. Bein *et al.* [16] observed the same orientation preference in their crystallization studies of MOFs. In their studies, organothiol-based COOH-terminated and OH-terminated SAMs were immersed at room temperature into an aged (eight days) and filtered mother solution from the solvothermal synthesis of HKUST-1 [16].

The deposition of organic layers using such sequential processes has been reported previously, like for deposition of multilayers of organosulfur/Cu compounds and for the deposition of ionic polymers [23,24], and also in our early work on the sequential deposition of Zn_x_(btc)_y_, which has a different structure from HKUST-1 [25]. However, in this case only disordered polymeric structures were obtained in contrast to SURMOFs with a well defined diffraction pattern.

**Figure 4:**
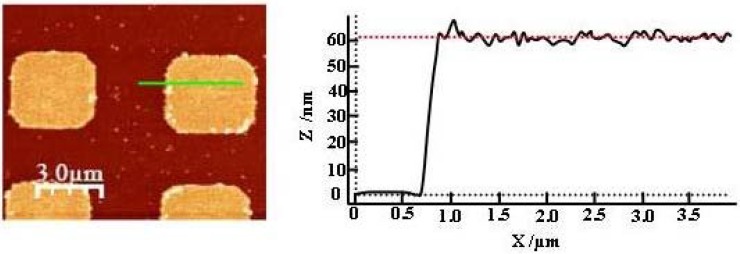
AFM image of Cu_3_(btc)_2_.xH_2_O MOF (45 cycles) grown on a SAM laterally patterned by microcontact printing (µCP) consisting of COOH-terminated squares and CH_3_-terminated stripes (left), corresponding height averaged profile (calculated over the whole area (right) [37].

Quantitative AFM measurements have allowed following the growth of HKUST-1 SURMOF on the COOH-terminated surface and studying their morphological characteristics. The results in Figure 4 verify the selective growth of the HKUST-1 on a laterally patterned substrate by microcontact printing (µCP) [26], consisting of COOH-terminated squares and CH_3_-terminated stripes and the homogeneity of the deposited layers. This shows the validity of the LBL preparation procedure employed and its capacity to fabricate high quality MOFs films on surfaces in contrast to the other deposition methods [15,16,27].

### 2.2. Synthesis and growth of layer based MOFs (LBMOFs)

**Figure 5:**
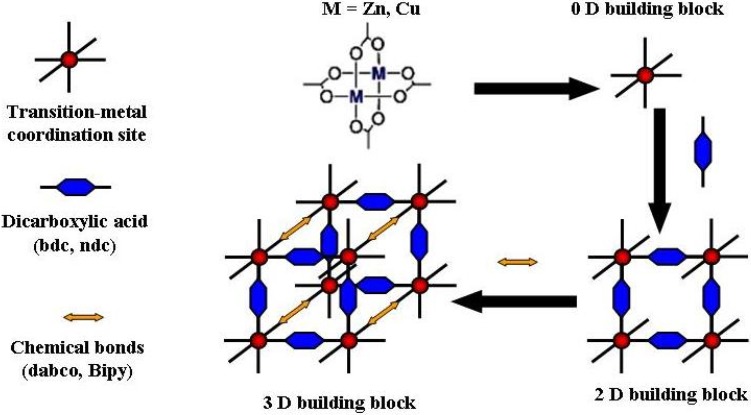
Schematic representation for the synthesis and formation of the 2D and 3D LBMOFs.

**Figure 6:**
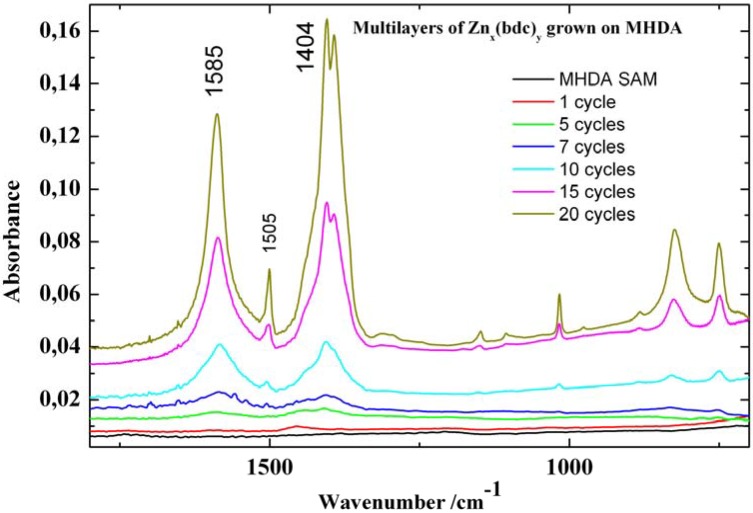
IRRAS spectra of different cycles of Zn_x_(bdc)_y_ MOF grown on a MHDA SAM.

Layer based MOFs (LBMOFs) are one class of MOFs, which are generally built up by bridges of dicarboxylate ligands [28,29,30]. In the case of the linear dicarboxylate bridges, two dimensional (2D) lattices are constructed, and infinite linear micro-pores are created by stacking the 2D lattices [M_2_(OOC-L-COO)_2_]_n_ (2D) (M = Cu, Zn, Co, Ru and L = organic ligand), as shown in Figure 6 [30]. Three-dimensional building blocks are generated by using linear connectors like 1,4-diazabicyclo[2.2.2]octane) (dabco) to connect the 2D lattices as shown in Figure 5 [29].

As the LBMOFs structure shows, they have a COOH and pyridine donors that are bound orthogonally to the metal coordination site, which makes it most interesting to mimic the two types of donors and see whether the two different SAM terminations (*i.e.,* a COOH and pyridine terminations) could result in a complementary arrangement of the layers.

Firstly, we have studied the growth of both (Cu_x_(bdc)_y_) and (Zn_x_(bdc)_y_) (bdc = benzene dicarboxylate) multilayers on a COOH-terminated surface made from MHDA SAM without using any linear connectors. The layers were fabricated by immersing the COOH-terminated surface into an ethanolic solution of Zn_2_(CH_3_COO)_2_.H_2_O (Zn(Ac)_2_) and then in ethanolic solution of H_2_bdc (H_2_bdc = benzene dicarboxylic acid). The IRRAS results show that subsequently adding Zn(Ac)_2_ and H_2_bdc leads to a stepwise deposition of Zn_x_(bdc)_y_ multilayers and an increase in the thickness of the multilayers with the increase of the deposition cycles (Figure 6). The SPR data also support this finding.

**Figure 7:**
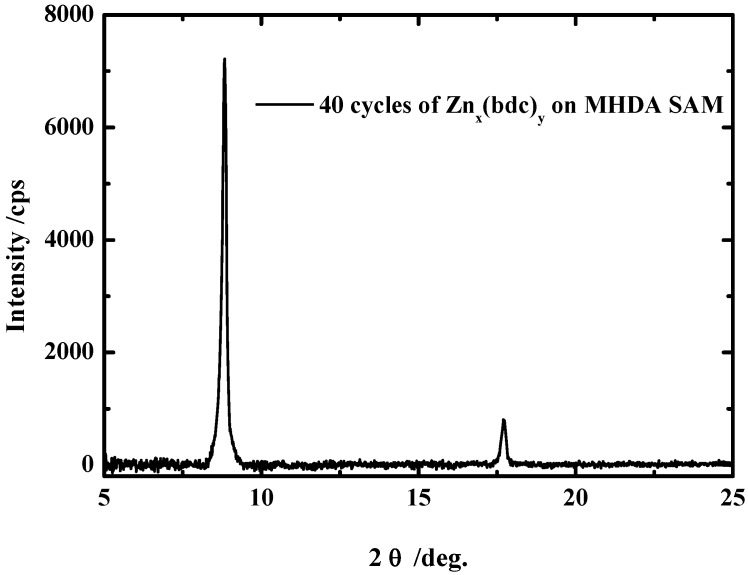
Out-of-plane XRD data for 40 cycles of Zn_x_(bdc)_y_ MOF grown on a MHDA SAM.

A typical diffraction scan for a 40 cycles Zn_x_(bdc)_y_ multilayers is shown in Figure 7. This out-of-plane diffraction scan clearly demonstrates the presence of a highly ordered and preferentially oriented crystalline material, but so far we do not know what the real structure is since no XRD data of such system where available for comparison [29]. The same procedure was applied to grow the Zn_x_(bdc)_y_ on the pyridine terminated SAM made from 4,4-pyridyl-benzenemethanethiol (PBMT). In contrast to the COOH terminated SAMs, no XRD data were obtained, which shows again the importance of the surface termination for the growth. The same results were obtained for the growth of Cu_x_(bdc)_y_ layers on both surfaces.

We then tried to grow the 3D LBMOFs by using a linear connector like 1,4-diazabicyclo[2.2.2]octane (dabco) to connect the 2D layers. [Zn_2_(bdc)_2_(dabco)] was selected as a typical example of these 2D zinc carboxylate layers, which are linked together by dabco via the vacant coordination sites at the Zn^2+^ centres of the zinc carboxylate layers to form a 3D LBMOF [31], exhibiting a two-dimensional open framework of interconnected Zn_2_(bdc)_4_ paddlewheel units [31].

The deposition of microcrystalline, phase pure [Zn_2_(bdc)_2_(dabco)] under solvo-thermal conditions on alumina and on silica surfaces modified with self assembled organic monolayers has been studied using the *in situ* crystallization method [32]. The growth of [Zn_2_(bdc)_2_(dabco)] was found to be not surface selective at all and only densely packed coatings were obtained on silica and on alumina surfaces.

In the present case, we studied the growth of [Zn_2_(bdc)_2_(dabco)] on Au(111) surfaces modified with self-assembled organic monolayers terminated by either with COOH or pyridine units, in order to study the effect of the SAM functionality on the growth orientation of [Zn_2_(bdc)_2_(dabco)] LBMOF. Both surfaces were immersed by turns in the two different solutions of Zn(Ac)_2_ and of the organic ligands (H_2_bdc + dabco) mixture, which were kept separated in two beakers with intermittent rinsing.

**Figure 8:**
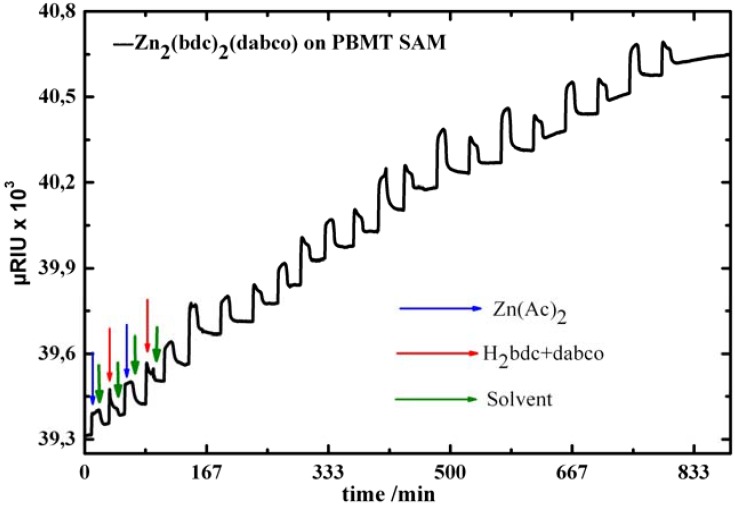
SPR signal as a function of time recorded *in situ* during sequential injections of Zn(Ac)_2_, ethanol , and mixture of H_2_bdc+dabco in the SPR cell containing BPMT SAM.

**Figure 9:**
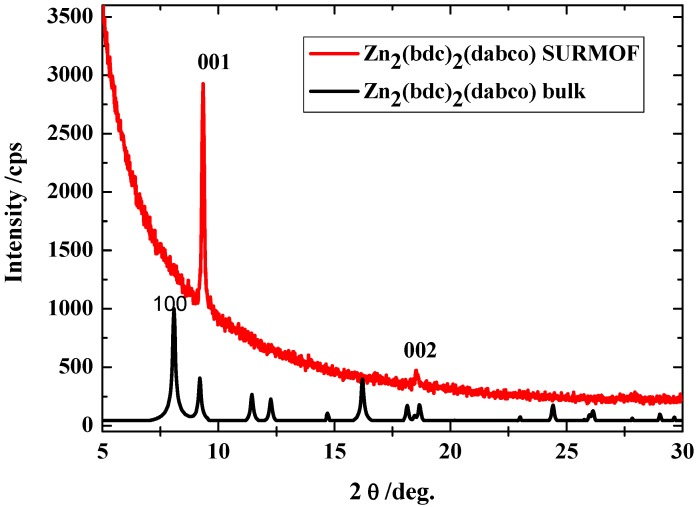
Out-of-plane XRD data for a [Zn_2_(bdc)_2_(dabco)] MOF sample (40 cycles) grown on a pyridine terminated SAM (red). The XRD for the bulk (black) is shown for comparison.

As evidenced by the SPR data shown in Figure 8, the LBL synthesis led to a stepwise growth of an organic/inorganic multilayers on the substrate. Figure 9 clearly shows that after an initiating period, each step of growth leads to roughly the same amount of deposited material.

The XRD results in Figure 9 show the corresponding diffraction pattern recorded in the out-of-plane diffraction modus for the SURMOF material of the likely composition [Zn_2_(bdc)_2_(dabco)] with 40 deposition cycles deposited on the pyridine-terminated organic surface. The two sharp diffraction peaks indicate the presence of highly oriented material that has the same structure like the bulk materials and is oriented along the (001) direction. The growth of [Zn_2_(bdc)_2_(dabco)] on MHDA, a COOH terminated SAM, leads to the growth of MOF material of the likely composition [Zn_2_(bdc)_2_(dabco)], but with mixed orientations (not shown here), which indicates that the BPMT surface is the most suitable surface for the LBMOFs growth.

**Figure 10:**
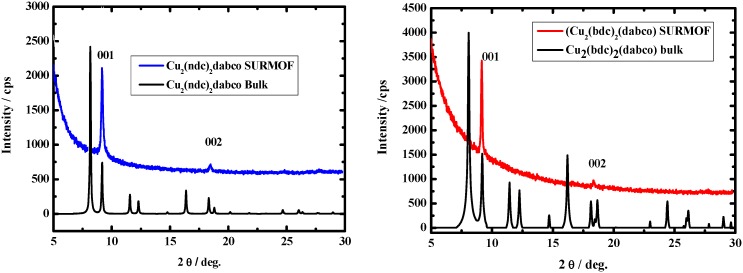
Out-of-plane XRD data for a [Cu_2_(ndc)_2_(dabco)] MOF sample (left) and [Cu_2_(bdc)_2_(dabco)] (right) grown on a pyridine terminated SAM. The XRD of for the bulk is shown for comparison.

Different other types of these LBMOFs like ([Cu_2_(bdc)_2_(dabco)] and [Cu_2_(ndc)_2_(dabco)] (ndc=1,4**-**Naphthalenedicarboxylic acid) have been grown using the same approach on the pyridine terminated SAM. The XRD data shows clearly that they all also grow highly oriented (Figure 10).

We then tried to grow another type of the LBMOFs, namely the [Zn(bdc)(4,4′-Bipy)_0.5_] MOF, which is synthesized by replacing the dabco connector with the bipy (bipy = 4,4′- bipyridine) ligand.

**Figure 11:**
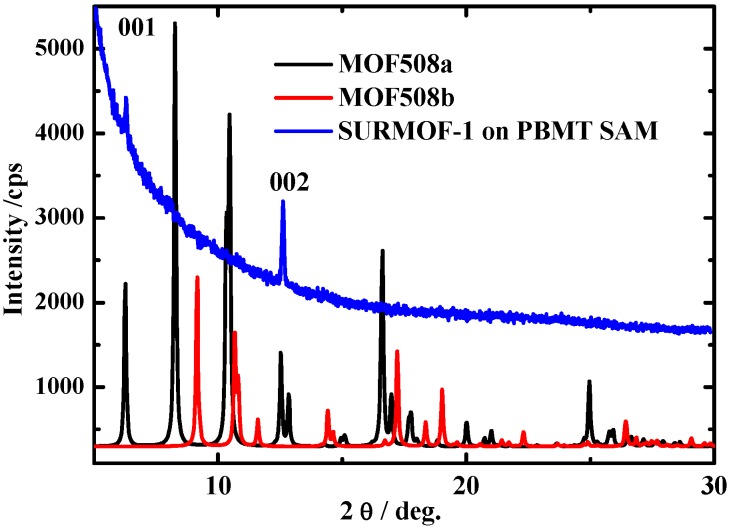
Out-of-plane XRD pattern (blue) for a [Zn(bdc)(4,4′-Bipy)_0.5_] SURMOF-1 sample (40 cycles) grown on a pyridine terminated SAM from PBMT. The XRD patterns for the two possible bulk phases are shown for comparison [34].

Figure 11 shows the corresponding diffraction pattern recorded in the out-of-plane diffraction modus for MOF material of the likely composition [Zn(bdc)(4,4′-Bipy)_0.5_] with 40 deposition cycles deposited on the pyridine-terminated organic surface. The two sharp diffraction peaks indicate the presence of highly oriented material with a large lattice constant. We assign the two diffraction peaks based on the corresponding XRD bulk data for the two different polymorphs of MOF-508, which are shown in the bottom of Figure 11. Clearly, the positions of the two Bragg peaks in the XRD data of the SURMOF fit only with MOF-508a.

However, when comparing the experimental XRD pattern for the SURMOF to a simulation of the diffraction pattern expected for an oriented MOF layer of the type MOF-508a on the surface, we see a major deviation: the relative intensities of the [100] and [200] peaks seen in the SURMOF XRD data differ greatly from those seen for MOF-508a [33]. The IR spectroscopy reveals that the constituents of this lattice are the same units as present in MOF-508a, the presence of solvent and other adsorbed species can be excluded.

Since the thickness of the SURMOF is too small to allow for a direct structure determination using XRD, we further characterized the SURMOF by determining its porosity. This was a difficult task due to a tiny amount of SURMOF material, but was done using a very sensitive measurement of the Kr-BET surface area [34]. A value of 627 ± 15 m^2^/cm^3^ for the inner surface from the Kr-BET, slightly more than half the Kr-BET value for the interpenetrated MOF-508a, 930 ± 15 m^2^/cm^3^. (The corresponding N_2_ BET value amounts to 821 m^2^/cm^3^) [34].

The fact that the BET surface per volume for the interpenetrated and desolvated MOF-508a and MOF-508b is less than double of the non-interpenetrated and solvent free framework of our SURMOF is expected, since the two interpenetrating networks in MOF-508a and MOF-508b will be so close in space that the inner surface available for Kr-adsorption will be less than double the value of a single framework. When converted to surface area per weight we obtain values of (1010 m^2^/g), substantially more than the value of 660 m^2^/g for the interpenetrated MOF-508a and MOF-508b.

The findings reported above demonstrate that our synthesis route yields a crystalline framework with the same composition and unit cell parameters as MOF-508a but with only about half the inner surface/volume area. The simplest explanation for these findings is that the SURMOF is the non-interpenetrated and solvent-free analogue of MOF-508a. A calculation of the corresponding XRD diffraction peak intensities reveals an excellent agreement [34].

We explain the suppression of the second, interpenetrating lattice in the SURMOF by the pyridine-terminated organic surface acting as nucleation template. The other sub-lattice does not match with this template, and it therefore cannot nucleate at the surface.

## 3. Experimental

### 3.1. SURMOFs preparation

**Figure 12:**
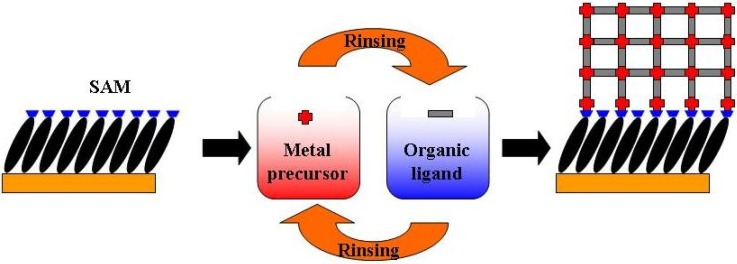
Schematic diagram for the step-by-step approach for the growth of the MOFs on substrates functionalized with SAMs. The approach is done by repeated immersion cycles first in solutions of the metal precursor and subsequently in the solution of organic ligand, after rinsing with the solvent in between.

The procedure for the preparation and growth of SURMOFs investigated here is schematically illustrated in Figure 12. Gold substrates (200-nm Au/2-nm Ti evaporated on Si wafers) were first functionalized with self-assembled monolayers (SAMs) [35]. The freshly prepared substrates were then immersed subsequently in a 1 mM of the metal precursor (M_2_(CH_3_COO)_2_.H_2_O (M = Cu or Zn)) ethanol solution for 30 minutes and in a 0.1 mM of the organic ligands ethanol solution for 60 minutes at room temperature. Between each step the substrates were rinsed with ethanol and dried in a nitrogen stream.

### 3.2. SURMOFs characterization

IRRAS data were recorded using a Biorad Excalibur FTIR spectrometer (FTS 3000) equipped with a grazing incidence reflection unit (Biorad Uniflex) and a narrow band MCT detector. A commercial surface plasmon resonance (SPR) system ((Reichert SR7000DC) was used to record the real-time kinetics adsorption to the organic surface [36]. A commercial quartz crystal microbalance with dissipation (QCM-D) system (Q-Sense E4 Auto) was used to record the real-time surface interactions with the organic surface. X-ray diffraction (XRD) data for out-of-plane conditions were measured using a laboratory (Cu Kα) source. Topographic AFM images were acquired both in contact and dynamic modes under ambient conditions using a commercial head and software from Nanotec [37].

## 4. Conclusion

In this work, a new LBL method for the synthesis and growth of SURMOFs on functionalized organic surfaces has been presented. The new LBL method showed that it is possible to control the growth of different types of MOFs thin films, which are listed in Table 1.

Through using different types of surface terminations it was also possible to control the SURMOF growth orientation. The SURMOF thin films produced using this method are very flat and homogenous, in contrast to the films produced by the *in situ* crystallization method. The kinetics of the thin film growth was also monitored *in situ* using SPR and QCM-D. It was also possible to demonstrate the validity of this method to synthesis new types of MOFs that are not accessible by bulk methods, as in the case of controlling the interpenetration in MOF-508.

**Table 1:** A list of the types of MOFs that have been synthesized using the layer-by-layer method and the type of SAM termination used for growth.

Inorganic coupling unit	Organic ligand	MOF	SAM termination
Cu(Ac)_2_	H_3_btc	Cu_3_(btc)_2_. xH_2_O	COOH, OH
Cu(Ac)_2_	H_2_bdc	Cu_x_(bdc)_y_	COOH
Cu(Ac)_2_	H_2_bdc+dabco	Cu_2_(bdc)_2_(dabco)	COOH, Pyridine
Cu(Ac)_2_	H_2_ndc+dabco	Cu_2_(ndc)_2_(dabco)	COOH, Pyridine
Zn(Ac)_2_	H_2_bdc	Zn_x_(bdc)_y_	COOH
Zn(Ac)_2_	H_2_ndc+dabco	Zn_2_(ndc)_2_(dabco)	COOH, Pyridine
Zn(Ac)_2_	H_2_bdc+4,4′-Bipy	Zn(bdc)(4,4′-Bipy)_0.5_	Pyridine
